# Advances in understanding the tumor microenvironment of neuroendocrine prostate cancer

**DOI:** 10.3389/fonc.2026.1928713

**Published:** 2026-08-28

**Authors:** Nan Yao, Qixing Yang, Pengkang Chang, Yangcai Wang, Hao Li, Ronghua Wu, Ji Zheng, Weihua Fu

**Affiliations:** 1 Department of Urology, The Second Affiliated Hospital, Army Medical University, Chongqing, China; 2 The First Clinical Medical College, Nanchang University, Nanchang, Jiangxi, China

**Keywords:** cancer-associated fibroblasts, DLL3, lineage plasticity, neuroendocrine prostate cancer, spatial transcriptomics, targeted therapy, therapy resistance, tumor microenvironment

## Abstract

Neuroendocrine prostate cancer (NEPC) is an aggressive, treatment-refractory phenotype of advanced prostate cancer. This narrative review was informed by targeted searches of PubMed, Web of Science through May 2026. We classify evidence as Level A (human NEPC samples or clinical cohorts), Level B (NEPC-specific models), Level C (prostate adenocarcinoma or CRPC without NEPC resolution), or Level D (pan-cancer or non-prostate extrapolation), and apply these levels to central claims and a study-level evidence table. Direct human data support immune depletion in most NEPC tumors, with heterogeneous macrophage, fibroblast and lymphoid remodeling in selected cohorts; model studies support context-dependent cytokine, hypoxic, extracellular-matrix and metabolic effects on lineage plasticity. By contrast, CAF-derived lactate fueling NEPC, a dense collagen drug barrier, and NEPC-specific vascular permeability remain unvalidated. Most TME-directed treatments are preclinical or extrapolated, whereas DLL3-directed therapy has shown early activity in biomarker-selected disease. Longitudinal biopsies, spatial multi-omics, humanized models and biomarker-defined trials are needed to distinguish causal vulnerabilities from correlates of treatment-emergent lineage transition.

## Graphical Abstract

**Figure:**
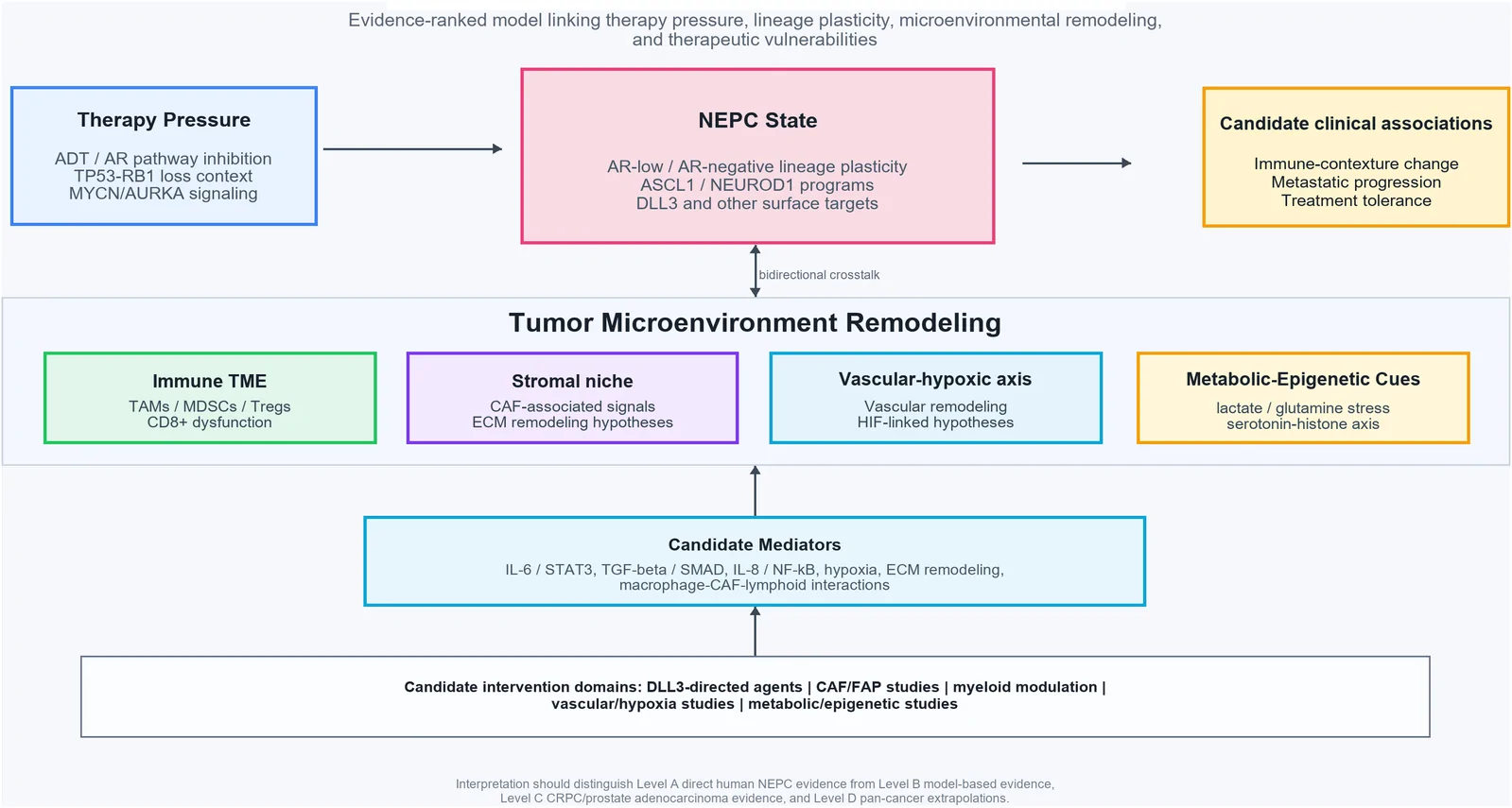
Evidence-ranked conceptual model linking therapy pressure, NEPC lineage plasticity, TME remodeling and candidate therapeutic vulnerabilities. Original figure prepared by the authors for this review; it should be interpreted as a synthesis rather than as a single experimentally validated pathway.

## 1 Introduction

### 1.1 Clinical significance of NEPC

According to GLOBOCAN 2022 estimates reported in CA: A Cancer Journal for Clinicians, prostate cancer accounted for approximately 1.47 million new cases and 397,000 deaths worldwide in 2022, ranking as the fourth most commonly diagnosed cancer overall and the second most common cancer among men (1). In the United States, the 2026 CA Cancer Statistics report projects 333,830 new prostate cancer cases and 36,320 deaths, making prostate cancer the leading newly diagnosed cancer among men in that setting (2). Recent CA prostate cancer statistics further indicate that US incidence has been rising again, particularly for advanced-stage disease, whereas mortality declines have slowed (3). NEPC is rare as a *de novo* entity but may emerge under therapeutic pressure in a subset of metastatic castration-resistant prostate cancer (mCRPC), especially after prolonged androgen receptor pathway inhibition (4–6). Therefore, epidemiological statements should be geographically qualified and should avoid describing prostate cancer as the most commonly diagnosed male malignancy worldwide without qualification.

Clinically, NEPC is characterized by rapid progression, early metastasis and a dismal prognosis. Patients typically exhibit low or only mildly elevated serum PSA concentrations, yet suffer severe constitutional symptoms, visceral metastases and paraneoplastic syndromes (4, 7). Median survival among patients with NEPC is usually less than 12 months, substantially shorter than that observed in prostatic adenocarcinoma (5, 6). The widespread adoption of ADT and next-generation hormonal agents has driven a marked rise in t-NEPC incidence, creating a major clinical challenge (5, 6, 8).

At the molecular level, NEPC cells are typically AR-negative and refractory to conventional endocrine therapies (5, 7, 9). Hallmarks of NEPC include high expression of neuroendocrine markers (CHGA, SYP, NSE), lineage-specifying transcription factors (ASCL1, NEUROD1) and silencing of AR signaling (6, 10). NEPC is also characterized by biallelic loss of the tumor suppressors TP53 and RB1, together with amplification of the oncogenes MYCN and AURKA (4, 7).

NEPC pathogenesis reflects the interaction of tumor-intrinsic lineage programs with therapy pressure and extrinsic signals. Genetic and epigenetic changes create competence for lineage switching, whereas stromal, immune, vascular and metabolic conditions may select or stabilize particular states (10–16). This review focuses on that interface rather than treating tumor-intrinsic targets as TME components.

### 1.2 Importance of the tumor microenvironment

The tumor microenvironment (TME) is a dynamic ecosystem of immune, stromal, vascular and extracellular-matrix compartments. During progression from prostate adenocarcinoma to treatment-emergent NEPC, androgen-receptor pathway inhibition first alters epithelial state and can remodel stromal and immune signaling; these changes may then reinforce lineage plasticity, immune dysfunction and treatment tolerance. The temporal order is incompletely resolved because paired longitudinal human biopsies remain uncommon (16–19).

Recent single-cell and spatial transcriptomic analyses have provided higher-resolution views of prostate cancer progression. A 2026 integrated atlas of 127 single-cell RNA-sequencing samples and 9 spatial transcriptomic profiles spanning healthy prostate to neuroendocrine carcinoma reported evolutionarily connected epithelial states and therapy-associated remodeling of macrophage, CAF and lymphoid compartments (19). These data strengthen the rationale for studying NEPC–TME interactions, but they should not be overinterpreted as proving that every proposed immune, vascular or stromal feature is uniquely NEPC-specific.

Defining TME contributions may guide therapeutic development, but the evidence is uneven. TME-modulating strategies have clinical relevance in other cancers and preclinical prostate-cancer systems, while NEPC-specific clinical validation is limited (18, 20–23). Co-targeting tumor cells and their environment remains investigational unless supported by a disease-specific biomarker and prospective trial (18, 23–26).

Biomarker terminology should be tied to intended use. Histology and neuroendocrine markers support diagnosis; outcome-linked FAP or immune signatures are candidate prognostic markers; DLL3 expression is a patient-selection marker for DLL3-directed trials; serial circulating or spatial measures may serve pharmacodynamic roles. A predictive claim requires evidence that treatment benefit differs by marker status, and these categories should not be used interchangeably (27–34).

Evidence-grading framework: Level A denotes human NEPC samples or clinical cohorts; Level B denotes NEPC-specific PDX, organoid, cell, co-culture, xenograft or genetically engineered models; Level C denotes CRPC or prostate adenocarcinoma without NEPC-resolved analysis; and Level D denotes pan-cancer or non-prostate extrapolation. Central claims below identify the highest applicable level parenthetically, and mixed evidence is described in the same sentence rather than through a global disclaimer. **Table 1** defines the operational criteria, and **Table 2** reports study-level PMID, cohort/model, sample size, NEPC definition, principal result and limitations.

**Table 1:** Evidence levels of NEPC-TME findings. Representative study-level references supporting the examples are provided in **Table 2**.

Evidence level	Operational definition	Examples in this review	Required claim language
Level A	Human samples or clinical cohorts explicitly annotated as *de novo* or treatment-emergent NEPC by histology, molecular criteria, or study-defined NEPC classification.	Human immunogenomics, scRNA/spatial studies, DLL3-positive NEPC trials, and molecularly annotated FAP data.	May support NEPC-specific association statements; causal language still requires interventional or longitudinal evidence.
Level B	NEPC PDX, organoid, cell-line, co-culture, xenograft, or genetically engineered models with neuroendocrine identity verified by the source study.	MCT4-dependent tumor-cell lactate secretion, EndoMT signaling, epigenetic/metabolic lineage programs, and DLL3-directed model studies.	Use ‘model-supported’ or ‘demonstrated in NEPC models’; do not imply validation in patients.
Level C	CRPC or prostate adenocarcinoma evidence without NEPC-resolved analysis.	CAF states, stromal MCT4/tumor MCT1 lactate shuttling, fibrosis, angiogenesis, and treatment-resistance mechanisms.	State the prostate-cancer context in the same sentence and use ‘may contribute’, ‘is consistent with’, or ‘candidate mechanism’.
Level D	Pan-cancer, non-prostate, or general TME evidence.	General macrophage-Treg feedback, collagen barriers, vascular permeability, and metabolic immunosuppression.	Label as extrapolation or hypothesis-generating; do not present as an established feature of NEPC.

Evidence level describes source proximity to NEPC, not study quality by itself. Level A can establish human association but not necessarily causality; direct causal language is reserved for NEPC-specific experiments demonstrating perturbation, necessity, sufficiency or therapeutic reversal.

**Table 2:** Study-level evidence map for claims central to this review.

Study	Cohort/model and NEPC definition	Sample size	Principal finding	Level	Major limitation
(19); PMID 41764551	Integrated scRNA/spatial atlas spanning healthy prostate to neuroendocrine carcinoma.	127 scRNA samples; 9 spatial profiles	Links epithelial state transitions with therapy-associated macrophage, CAF, and lymphoid remodeling.	A	Cross-sectional integration; NEPC-specific sample contribution is not separately quantified in the abstract.
(20); PMID 37223924	Retrospective prostate-cancer cohort; study-defined NEPC compared with other prostate cancers and SCLC.	170 patients; 230 RNA-seq; 104 WES; NEPC n=36	NEPC was predominantly immune depleted; 9/36 tumors were T-cell inflamed.	A	Retrospective sampling and heterogeneous prior therapy; association does not prove treatment response.
(31); PMID 38760413	Rapid-autopsy metastatic CRPC cohort with molecular NEPC classification and PDX validation.	Cohort size not stated in abstract; multiple PDX models	DLL3 and CEACAM5 were highest in NEPC, whereas TROP2 was lower in NEPC.	A/B	Post-mortem cohort and intrapatient heterogeneity; expression does not establish therapeutic efficacy.
(35); PMID 35292724	Open-label phase 2; histologic NEPC or aggressive-variant criteria.	15 men	Avelumab ORR 6.7%; the complete responder had MSI-high/MSH2-altered disease.	A	Single center, small cohort, and early termination during COVID-19.
(33); PMID 35052475	Prospective multicenter mCRPC cohort; NEPC-high/AR-low expression scores.	208 patients; high FAP n=21	High FAP expression associated with NE signaling and shorter survival.	A	Molecular rather than uniform histologic NEPC definition; observational association.
(36); PMID 35789834	Patient tumors containing adenocarcinoma and NEPC analyzed by scRNA-seq.	7 patients	Defined divergent routes from adenocarcinoma to NEPC and two transition signatures.	A	Small cohort; transcriptional inference does not establish TME causality.
(34); PMID 40689871	Phase 1b DeLLpro-300; metastatic *de novo* or treatment-emergent NEPC by histologic, genomic, or IHC criteria.	40 patients; DLL3-positive n=18	Tarlatamab ORR was 10.5% overall and 22.2% in DLL3-positive tumors.	A	Single-arm early-phase study; retrospective DLL3 testing and wide confidence intervals.
(37); PMID 37240308	Spatial profiling of coexisting *de novo* NEPC and AR-pathway prostate cancer.	1 patient	NEPC regions showed distinct lineage and fibrosis-associated TME signals.	A	Single case; hypothesis-generating only.
(38); PMID 40705968	scRNA-seq across PDX models representing five NEPC pathologies.	9 PDX models; 18,632 tumor cells	Resolved pathology-specific programs and limited clonal divergence in focal NED.	B	PDX models lack a fully intact human immune microenvironment.
(10); PMID 30287808	ADT-treated prostate models, xenografts, and correlative patient samples with neuroendocrine features.	Multiple assays; n varies by experiment	CREB-EZH2-TSP1 signaling connected neuroendocrine differentiation with angiogenesis.	B	Supports angiogenic remodeling, not vessel leakiness or NEPC drug-distribution pharmacology.
(39); PMID 40706636	EndoMT-induced HUVEC/LNCaP co-culture and signaling perturbation.	Assay-specific n	Endothelial GM-CSF promoted neuroendocrine features through STAT3 signaling.	B	*In vitro* model; not quantitative human NEPC vascular evidence.
(40); PMID 39024561	NE and Rb1/Trp53-deficient prostate cancer models.	Multiple models; assay-specific n	Notch activation shifted NE tumors toward a non-NE state and increased MHC/type I IFN programs.	B	Preclinical; magnitude and reversibility in patients are unknown.
(41); PMID 20215503	CR2-TAg transgenic neuroendocrine prostate tumor model with MMP perturbation.	Genotype- and assay-specific animal n	MMP-2 loss reduced tumor burden, metastasis, and vessel density; MMP roles differed.	B	Legacy GEMM may not represent treatment-emergent human NEPC.
(42); PMID 38654072	Multi-omics, organoid formation, and *in vivo* lineage tracing in NEPC development models.	Multiple models; assay-specific n	Zeb1-linked glycolysis and lactate-associated chromatin accessibility supported a transition state.	B	Tumor-intrinsic metabolic mechanism; does not establish CAF-derived lactate transfer.
(43); PMID 41416996	Genetically engineered and patient-derived NEPC xenograft models.	Multiple models; assay-specific n	Serotonin-driven histone serotonylation promoted lineage commitment; carbidopa inhibited growth.	B	Tumor-intrinsic and preclinical; clinical dose, selection, and efficacy remain unknown.
(44); PMID 40370549	*De novo* and treatment-induced NEPC PDX and organoid models.	Multiple models; assay-specific n	BRD4 inhibition with AZD5153 suppressed lineage-plasticity programs and tumor growth.	B	Preclinical and tumor-intrinsic; not evidence of a TME-targeted therapy.
(45); PMID 40102673	Antiandrogen-induced NEPC transition models with CRISPR and single-cell tracking.	Multiple models; assay-specific n	ZMYND8 inhibition blocked epigenetic programming of NEPC transdifferentiation.	B	Preclinical and tumor-intrinsic; human therapeutic window is unknown.
(46); PMID 29905005	Patient-derived NEPC xenografts, patient tumors, and MCT4 antisense perturbation.	Model panel; assay-specific n	NEPC showed elevated glycolysis and tumor-cell MCT4-dependent lactate secretion.	B	Does not test transfer of CAF-derived lactate into NEPC cells.
(47); PMID 24597899	Prostate epithelial/cancer cells, fibroblasts, xenografts, and human prostate tissues without NEPC resolution.	Multiple models; tissue n not stated in abstract	Stromal lactate supported tumor growth through tumor-cell MCT1.	C	Prostate-cancer evidence only; no NEPC-specific CAF-lactate validation.
(48); PMID 24886074	Hormone-naive radical-prostatectomy tissues; no NEPC resolution.	480 patients	High stromal MCT4 with tumor MCT1 associated with adverse pathologic features.	C	Localized adenocarcinoma cohort; observational and not applicable as direct NEPC evidence.
(49); PMID 37352863	Hormone-sensitive/CRPC CAF-state models and human correlations without NEPC-resolved analysis.	Multiple models; assay-specific n	ADT induced SPP1-positive myCAF switching and SPP1-ERK-mediated castration resistance.	C	CRPC stromal mechanism; NEPC-specific relevance remains to be tested.

Sample size denotes the main human cohort or number of models when reported in the verified abstract; experimental studies often use assay-specific sample sizes. Level A-D definitions are provided in **Table 1**.

### 1.3 Narrative literature search strategy

This narrative review was informed by targeted searches of PubMed, Web of Science for English-language articles published through May 2026 using combinations of neuroendocrine prostate cancer, tumor microenvironment, lineage plasticity, single-cell, spatial transcriptomics, immune microenvironment, cancer-associated fibroblasts, macrophages, DLL3 and therapy resistance. Human NEPC cohorts, clinical studies, single-cell or spatial datasets and mechanistic NEPC models were prioritized. Each central citation was checked against the claim and assigned Level A-D; prostate-cancer-general and pan-cancer evidence is identified as indirect in the relevant sentence. **Table 2** summarizes the primary studies most central to the synthesis. This was not a systematic review or meta-analysis, so no PRISMA screening, formal risk-of-bias scoring or quantitative synthesis was performed.

## 2 Cellular composition of the NEPC tumor microenvironment

### 2.1 Immune cells

**Figure 1:**
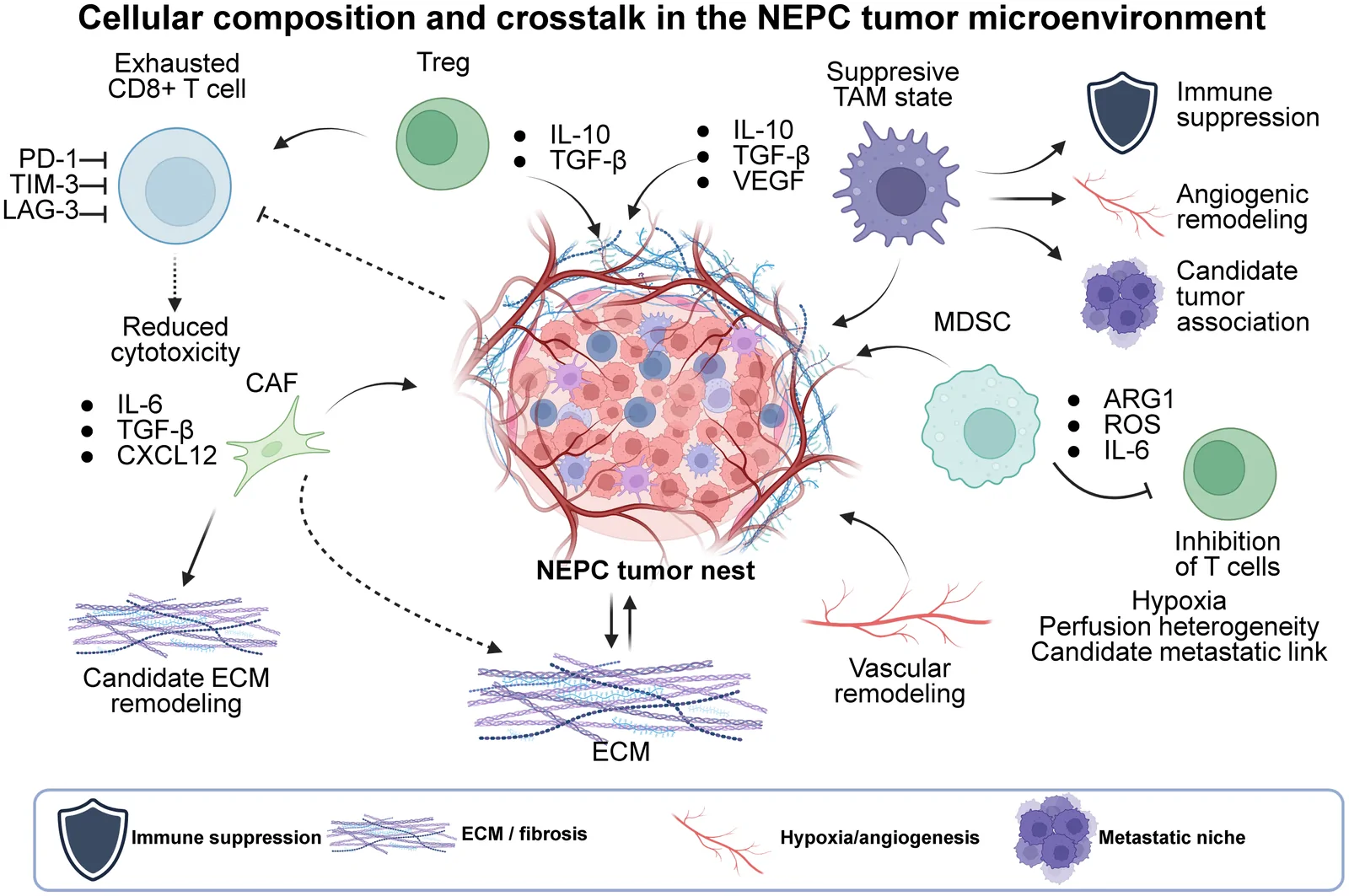
Candidate cellular composition and crosstalk in the NEPC tumor microenvironment. Functional labels such as ‘suppressive TAM state’ replace binary M1/M2 nomenclature. Human NEPC associations are distinguished from prostate-cancer and pan-cancer extrapolations; ECM and vascular links should not be interpreted as uniformly validated physical barriers in human NEPC.

Human immunogenomic and transcriptomic data indicate that many NEPC tumors are immune depleted, although a minority are T-cell inflamed (Level A) (19, 20, 30, 32, 50). Detailed mechanisms involving Tregs, suppressive macrophage states, MDSCs and checkpoints often derive from CRPC, prostate adenocarcinoma or pan-cancer studies (Levels C/D). **Figure 1** therefore presents candidate, evidence-ranked interactions rather than a uniform NEPC immune architecture.

CD8+ T-cell dysfunction is heterogeneous in advanced prostate cancer and NEPC. In a retrospective cohort of 36 NEPC tumors, 9 were classified as T-cell inflamed, whereas the remainder were immune depleted (Level A) (20). Other single-cell and immunogenomic studies describe reduced lymphoid support or exhaustion-associated programs during progression (19, 51–54). Direct spatial quantification of inhibitory receptors and cytokine output remains limited, and immune-checkpoint inhibitor monotherapy has little documented benefit outside biomarker-selected contexts such as MSI-high disease (35, 55, 56).

Tregs may suppress effector activity through IL-10/TGF-beta signaling and IL-2 consumption, but direct NEPC-specific quantification is sparse (Levels C/D) (17, 20, 32). Treg depletion or functional inhibition is investigational in NEPC (57).

Macrophage-enriched niches are observed in aggressive prostate cancer and NEPC-associated remodeling, but binary M1/M2 terminology does not capture the transcriptional and functional diversity of these cells. We therefore refer to defined features, such as TGF-beta-, IL-10- or VEGF-associated suppressive macrophage states. Human studies support association with NEPC context (Level A), while most causal and therapeutic claims remain model-based or extrapolated (Levels B-D) (19, 20, 32, 55, 58).

MDSCs are plausible contributors to immune evasion, but direct human NEPC evidence is sparse (Levels C/D). In prostate-cancer and general tumor models, these cells can reduce T-cell activity through reactive oxygen species and arginine depletion (59–62). Their prevalence, state and therapeutic relevance require prospective validation in annotated NEPC cohorts (50, 63–68).

Reciprocal regulation among Tregs and suppressive macrophage programs is well supported in pan-cancer literature (Level D) (20, 69–74). Whether the same feedback loops are necessary for immune dysfunction in human NEPC has not been demonstrated. Spatial profiling and perturbation studies should test these interactions rather than infer them from cell abundance alone.

### 2.2 Cancer-associated fibroblasts

CAFs are an important stromal compartment in NEPC-associated tissue, although claims that they are the dominant stromal population require quantitative validation across human cohorts (19, 33, 75–78).

FAP expression is associated with neuroendocrine transcriptional features and adverse outcome in a molecularly annotated mCRPC cohort (Level A) (33). Broader claims of uniformly greater collagen deposition or extensive fibrosis in NEPC remain unsupported by quantitative histology. Integrin-FAK-SRC and YAP/TAZ mechanotransduction is biologically plausible but is drawn mainly from prostate-cancer and pan-cancer fibrosis studies (Levels C/D) (12, 18, 39, 79, 80).

CAFs are one potential source of IL-6 and TGF-beta, but both cytokines are also produced by other stromal, immune and tumor-cell populations. IL-6/JAK/STAT3 and TGF-beta/SMAD signaling can induce neuroendocrine or EMT-associated programs in experimental systems (Levels B/C) (39, 80, 81). HGF and FGF effects are supported largely outside NEPC (Levels C/D) (82, 83).

Prostate adenocarcinoma studies support a stromal-tumor lactate shuttle in which fibroblast MCT4 expression is coupled to tumor-cell MCT1 expression. This was demonstrated in co-culture and xenograft experiments and associated with adverse features in prostatectomy cohorts of 480 and 535 patients (Level C) (47, 48). Separately, NEPC PDX and patient-tumor models show elevated tumor-cell glycolysis and MCT4-dependent lactate secretion (Level B) (46). These evidence streams should not be conflated: direct evidence that CAF-derived lactate fuels NEPC progression is lacking, and the mechanism remains a candidate for NEPC-specific testing.

Single-cell studies describe myofibroblastic, inflammatory and antigen-presenting CAF states, but dedicated NEPC analyses are limited (38, 84). Proposed subtype functions are drawn mostly from broader tumor atlases (Levels C/D) (77, 82, 85), so selective CAF targeting remains a research objective rather than an established opportunity.

CAFs can arise from resident fibroblasts and other mesenchymal or endothelial sources, but ontogeny is context dependent (26, 80, 86–88). Linking origin to function in NEPC will require lineage-resolved human data and models.

### 2.3 Vascular and hypoxic remodeling

NEPC-relevant models link neuroendocrine differentiation with angiogenic remodeling, including ADT-activated CREB-EZH2-TSP1 signaling (Level B) (10, 89). Human endothelial composition and vessel structure in NEPC remain poorly quantified. Thus, vascular remodeling and hypoxia are candidate features, while claims of uniformly abnormal NEPC vasculature require cohort-specific evidence (79, 90–93).

Hypoxia can promote neuroendocrine and EMT-associated programs in prostate-cancer models through HIF signaling (Levels B/C) (49, 55, 88, 94, 95). These data support a possible vascular-hypoxic contribution to lineage plasticity, but they do not establish impaired drug delivery as a general property of NEPC.

Anti-angiogenic therapy is not established for NEPC. Bevacizumab has shown limited benefit in small or mixed clinical settings (96, 97), and ADT-associated hypoxia has been linked to neuroendocrine features in prostate models (40, 89, 98). Biomarker-defined studies should directly measure perfusion, permeability and response before vascular normalization is considered an NEPC strategy.

## 3 Candidate TME-associated influences on neuroendocrine differentiation

### 3.1 Cytokine network regulation

The cytokine milieu may influence neuroendocrine transdifferentiation. CAFs and macrophages can contribute cytokines, but epithelial, endothelial and other immune populations are also sources, and their relative contributions in human NEPC remain unresolved (99, 100).

IL-6 can activate JAK/STAT3 and induce neuroendocrine transcriptional programs in relevant experimental systems (Levels B/C) (101, 102). The evidence supports IL-6/STAT3 as one candidate axis, not a universal or sole driver.

TGF-beta activates SMAD2/3-dependent EMT and neuroendocrine-associated programs in prostate-cancer models (49, 80, 103). Because TGF-beta is produced by multiple stromal, immune and tumor-cell populations, it should not be attributed predominantly to CAFs and macrophages without source-resolved NEPC data. Its association with aggressiveness is context dependent (Levels B/C).

IL-8 can activate NF-kappaB and has been linked to stemness-associated programs (104, 105). IL-8-mediated stabilization of HIF-1alpha through PI3K/AKT or MAPK signaling has primarily been demonstrated in experimental and non-NEPC systems (Levels C/D) (106, 107); its role in human NEPC remains to be tested.

These pathways may interact, but the architecture and persistence of any feedback circuit in human NEPC are not yet defined (18, 101).

### 3.2 Extracellular matrix remodeling

ECM remodeling may shape differentiation, invasion and treatment response, but the evidence spans distinct contexts (80, 108). CAF-derived matrix and stiffness-dependent signaling are supported in prostate-cancer and general tumor models (Levels C/D), while MMP perturbation provides NEPC-model evidence (Level B) (18, 41, 49, 81, 105, 109). Quantitative human NEPC measurements remain limited.

Specific ECM components should be interpreted cautiously. The cited literature does not establish that laminin promotes differentiation in human NEPC; laminin-integrin effects are therefore treated as indirect experimental context rather than an NEPC-specific mechanism (81). Hyaluronan-CD44 signaling can affect migration and invasion across tumor types (Level D) (110, 111).

Matrix stiffness can regulate stemness, invasion and YAP/TAZ signaling in prostate-cancer or pan-cancer models (Levels C/D) (112–114). Collagen organization and interstitial mechanics may also influence immune access or drug distribution, but direct evidence in human NEPC is insufficient (115–119). LOX, integrin/FAK and other ECM-directed interventions remain preclinical hypotheses.

### 3.3 Metabolic reprogramming

Metabolic reprogramming in NEPC should be separated into tumor-intrinsic and microenvironmental mechanisms. Tumor-cell glycolysis, MCT4-dependent lactate secretion, Zeb1-associated lactylation and serotonin-driven histone serotonylation are supported in NEPC models (Level B) (42, 43, 46). By contrast, CAF-to-tumor lactate shuttling is supported in non-NEPC prostate cancer (Level C) (47, 48), and direct transfer into NEPC has not been shown.

Other TME-associated metabolic stresses include lactate accumulation, glucose competition and glutamine use (42, 43, 120–124). These mechanisms are mostly Levels C/D unless the cited experiment resolves NEPC. Terms such as ‘glutamine addiction’ require direct dependency and rescue experiments in an NEPC model.

**Figure 2:**
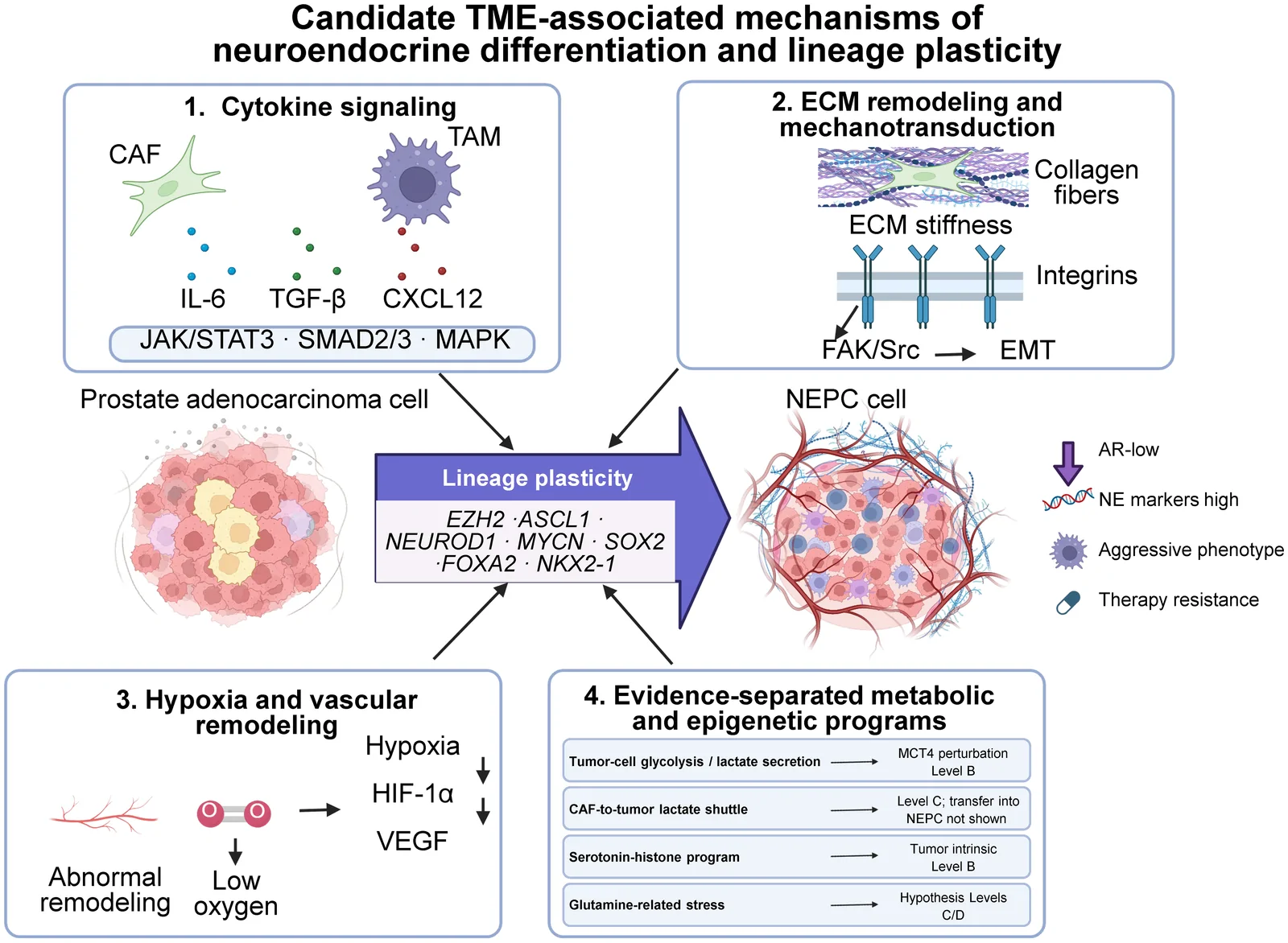
Candidate TME-associated influences on neuroendocrine differentiation and lineage plasticity. The hypoxia arrows were repositioned to avoid overlap, and the panel now uses ‘vascular remodeling’ rather than ‘vascular dysfunction.’ The metabolic panel separates NEPC tumor-cell MCT4 perturbation (Level B), non-NEPC CAF-to-tumor lactate-shuttle evidence (Level C), tumor-intrinsic serotonin signaling (Level B), and glutamine-related hypotheses (Levels C/D). Direct CAF-to-NEPC lactate transfer has not been shown, and the links should not be read as a proven linear pathway.

LDH, GLS, IDO and related interventions may modulate metabolic or immune programs in selected models, but NEPC-specific clinical evidence is absent. They are combination hypotheses, not established therapies. **Figure 2** summarizes the mixed evidence and explicitly separates Level B tumor-cell metabolism from Level C stromal lactate evidence.

## 4 Candidate TME-associated mechanisms of therapy resistance

### 4.1 Immunotherapy response

Limited response to immune-checkpoint blockade may reflect both tumor-intrinsic and TME-associated features, including reduced antigen-presentation programs, lineage-plastic states and suppressive myeloid or regulatory compartments (20, 124–126). Human NEPC data establish heterogeneity rather than a universal immune phenotype (Level A); most mechanistic links remain Levels C/D. Combinations should be selected by biomarkers and tested prospectively (36, 127).

Tregs, macrophage states and MDSCs can suppress T-cell activity through IL-10, TGF-beta, ARG1, reactive oxygen species and arginine depletion in relevant prostate-cancer or general models (5, 29, 36, 65, 128, 129). Their relative contribution to NEPC treatment response is unproven and requires spatial, treatment-linked biopsies.

Many NEPC tumors show reduced antigen-presentation programs, including decreased MHC-I expression, whereas a minority retain T-cell-inflamed features (Level A) (20, 36, 40). PD-L1 and PD-L2 expression occurs in subsets and should not be treated as universal (20, 130, 131). These observations may help explain limited immunotherapy activity but do not by themselves establish causal resistance.

Glucose competition, lactate accumulation and acidosis can impair T-cell function in general tumor systems (Levels C/D) (132–136). Whether these metabolic constraints are quantitatively dominant in NEPC is unknown.

### 4.2 Chemotherapy response

The TME may contribute to chemotherapy response through survival signaling, matrix mechanics and hypoxia, but direct NEPC evidence is limited (mainly Levels C/D). HGF/c-MET and FGF pathways have been implicated in therapy resistance outside NEPC (137–146), and ECM or vascular effects require disease-specific pharmacologic validation. These observations motivate experiments; they do not establish that TME targeting restores NEPC chemosensitivity.

CAF-derived HGF and FGF can activate PI3K/AKT or MAPK survival pathways in non-NEPC models (137–139, 144–146). They represent candidate chemoresistance mechanisms in NEPC, not demonstrated key drivers.

Collagen and fibrosis can restrict macromolecule transport or raise interstitial pressure in pan-cancer models (Levels C/D) (117–119, 140). References 148 and 149 do not quantify dense collagen networks in human NEPC. Accordingly, a physical barrier is biologically plausible, but NEPC-specific collagen architecture, pressure and intratumoral drug distribution remain research gaps.

Hypoxia can alter apoptosis and DNA-damage responses (142, 147). Its effect on DNA repair depends on the pathway, exposure duration and treatment context: suppression of selected repair programs may produce genomic instability or resistance, but can also increase sensitivity to particular DNA-damaging agents. This should not be reduced to a universal hypoxia-induced resistance mechanism.

### 4.3 Targeted-therapy response

TME-associated signaling and transport may influence targeted-therapy response, but current evidence is largely extrapolated (Levels C/D) (148–154). Candidate mechanisms include perfusion heterogeneity and growth-factor-mediated bypass signaling. Neither impaired delivery nor benefit from TME co-targeting has been established across NEPC.

NEPC-relevant models support angiogenic remodeling, including the CREB-EZH2-TSP1 axis (Level B) (10). They do not demonstrate intrinsically ‘leaky’ NEPC vessels or lower intratumoral drug concentrations. References 159 and 160 address general vascular monitoring and engineered nanoparticle-induced leakiness, respectively, and are not NEPC pharmacology studies. Direct measurements of permeability, perfusion, interstitial pressure and spatial drug distribution are needed.

CAF-derived FGF, IGF and other signals can activate bypass pathways in non-NEPC systems (153, 155, 156). Such redundancy may shorten response in selected contexts, but the claim that it precludes sustained response in NEPC has not been tested.

**Figure 3:**
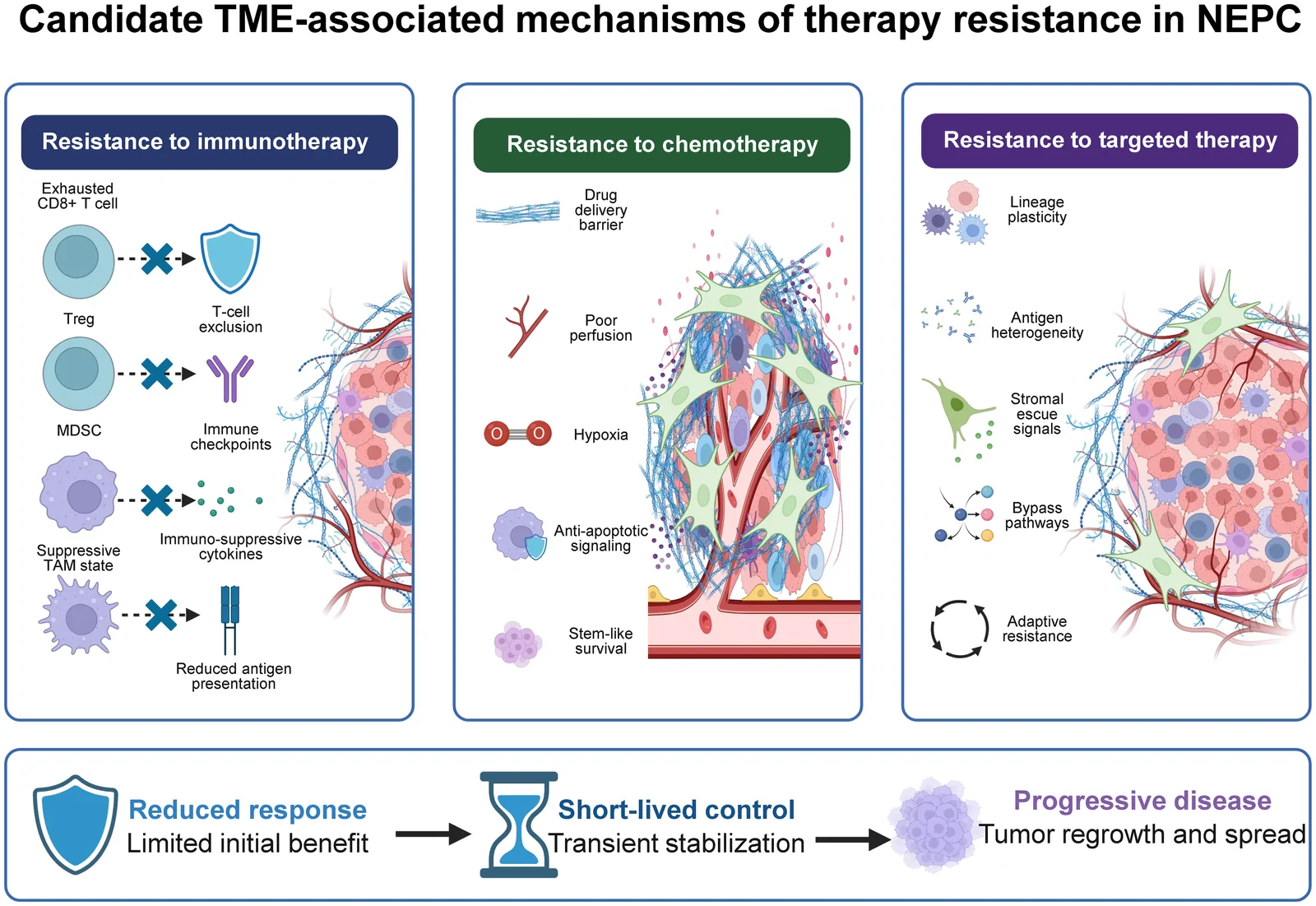
Candidate TME-associated factors related to therapy response in NEPC. The revised labels use functional macrophage-state terminology and ‘reduced antigen presentation’ rather than treating MHC-I loss as universal. The transport constraint, stromal signaling and clinical outcomes are presented as candidate associations; the diagram does not imply a TME-specific causal sequence. The pathways combine NEPC observations with Levels C/D extrapolation and should not be read as proven causes of resistance.

CAF-associated signals may maintain stem-like states in selected models (151, 157). Whether this process causes targeted-therapy resistance in NEPC, and whether its inhibition reverses resistance, remains unresolved. **Figure 3** presents these mechanisms as candidates.

## 5 Candidate TME-directed therapeutic strategies

**Figure 4:**
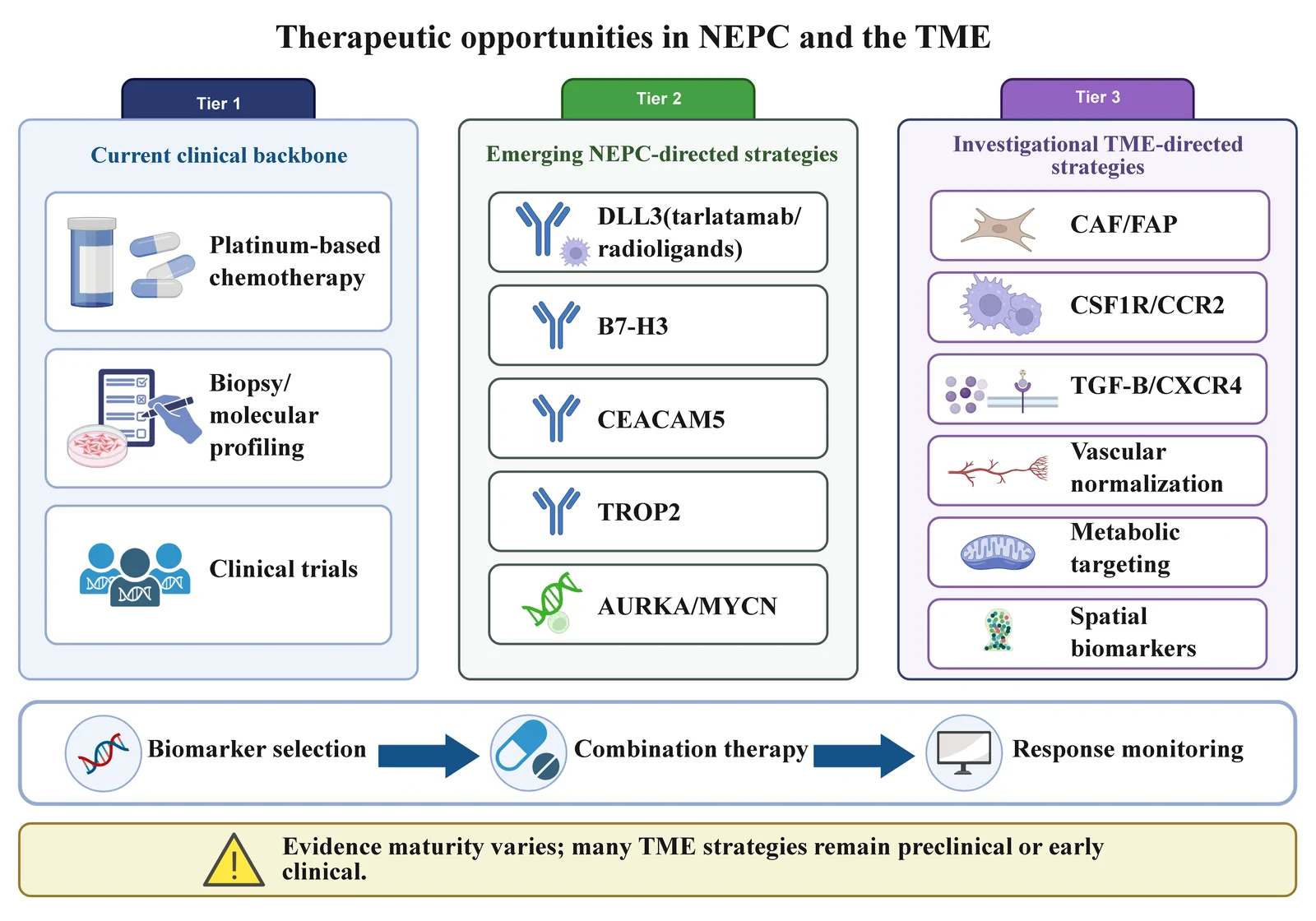
Therapeutic opportunities in NEPC and the TME. Major labels were enlarged for readability. The tiers separate current management, tumor-cell-directed NEPC strategies and investigational TME approaches; DLL3 and FAP are shown as patient-selection candidates, while response monitoring is pharmacodynamic. Tier placement reflects evidence maturity, not guideline recommendation.

An evidence-ranked overview of current, emerging, and investigational therapeutic opportunities in NEPC and the TME is shown in **Figure 4**.

### 5.1 Immune microenvironment modulation

Immune-TME strategies remain clinically unproven for most NEPC patients. Avelumab monotherapy had limited activity in a 15-patient NEPC/aggressive-variant trial, with a durable complete response in MSI-high disease (Level A) (35). CAF, macrophage, TGF-beta, IDO or A2AR modulation is supported mainly outside NEPC (Levels C/D) (158–168).

Combining checkpoint blockade with CAF- or TGF-beta-directed treatment is hypothesis-generating. Physical and cytokine-mediated T-cell constraints are documented in other tumors, but efficacy must be tied to the disease model studied (49, 126, 169–171).

TAM-directed strategies represent another active but still investigational avenue. CSF-1R inhibitors, CCR2 inhibitors and macrophage-reprogramming approaches can reshape myeloid compartments in selected preclinical or early clinical settings (170–173). For NEPC, these approaches should be framed as combination hypotheses pending disease-specific biomarker and efficacy data.

Metabolic-checkpoint blockade is a rapidly emerging field, but NEPC-specific clinical evidence remains minimal. IDO, adenosine-A2AR and related pathways can suppress T-cell activity in cancer, yet their relevance to NEPC should be considered hypothesis-generating unless supported by NEPC-specific models, biomarkers or trials (166, 174, 175).

### 5.2 Stromal cell-targeted therapy

CAF and stromal interventions are investigational in NEPC. FAP expression, IL-6/TGF-beta signaling, LOX/FAK mechanics and vascular normalization provide distinct hypotheses with different evidence levels (33, 41, 152, 167, 168, 176–181). Most efficacy data arise from pan-cancer, prostate adenocarcinoma or CRPC systems rather than NEPC trials.

FAP has several possible biomarker roles that should not be conflated. In molecularly annotated mCRPC, higher FAP expression was associated with neuroendocrine features and worse survival, supporting a candidate prognostic role (33). FAP imaging or expression may also serve as a patient-selection or pharmacodynamic marker in future trials, but it does not establish that FAP-directed drugs are effective or safe in NEPC (167, 168).

IL-6/JAK/STAT3 and TGF-beta signaling may influence lineage and immune programs, but blockade remains clinically unvalidated in NEPC. Siltuximab targets IL-6 rather than the IL-6 receptor. Any trial should define pathway activity, intended biomarker use and pharmacodynamic endpoints prospectively (180–182).

Tumor-intrinsic serotonin, BRD4 and ZMYND8 programs are retained only where they clarify how therapy pressure and microenvironmental selection intersect with lineage plasticity (43–45). They are not classified as TME-directed treatments and are summarized separately from stromal or immune interventions.

### 5.3 Interface between microenvironmental selection and tumor-intrinsic lineage plasticity

DLL3-directed radiopharmaceutical and radioimmunotherapy studies provide NEPC-model evidence (Level B) (183, 184). These agents target a tumor-cell surface antigen rather than the TME and remain preclinical.

DLL3-directed therapy is clinically more mature than most TME interventions. In the phase 1b DeLLpro-300 study, tarlatamab produced an objective response rate of 10.5% overall and 22.2% among DLL3-positive tumors (Level A) (34). DLL3 therefore functions as a patient-selection marker for this strategy, but activity is preliminary and confined to a subset.

### 5.4 Exploratory directions

Extracellular vesicles, microbiome modulation, nanomedicine and artificial-intelligence tools are retained as a brief exploratory agenda. Their current support is primarily pan-cancer, prostate-cancer-general or preclinical (Levels C/D) (185–201). Potential uses include studying intercellular cargo, systemic immune modulation, drug-delivery heterogeneity and spatial biomarker discovery; none should be presented as a near-term NEPC therapy.

## 6 Current research controversies and challenges

### 6.1 Limitations of preclinical models

Most current TME studies rely on cell lines or murine models that fail to fully recapitulate the heterogeneity and temporal dynamics of the human NEPC TME. Although organoid and humanized mouse models offer superior fidelity, they remain constrained by high cost, protracted experimental timelines, and incomplete immune reconstitution (202, 203). The pronounced inter- and intra-patient heterogeneity and rapid clonal evolution of NEPC impair the ability of existing models to capture authentic TME landscapes and therapeutic responsiveness (204, 205).

### 6.2 Mechanisms underlying heterogeneous response to immunotherapy

Reported response rates to checkpoint blockade remain modest. Candidate explanations include immune depletion in most tumors, reduced antigen-presentation programs in many cases and suppressive multicellular states (5, 20, 36, 125). These features are heterogeneous and should be tested as prognostic, predictive or pharmacodynamic biomarkers according to their intended use.

### 6.3 Therapeutic challenges posed by CAF heterogeneity

CAFs comprise multiple functional states with tumor-promoting or restraining effects. Nonselective depletion could remove beneficial states or cause toxicity, while NEPC-specific state markers and intervention data are lacking (206, 207).

### 6.4 Challenges in translating metabolic research

Metabolic pathways are plastic and shared with normal tissues. The principal translational challenges are patient selection, target-specific pharmacodynamic measurement, compensatory pathway use and systemic toxicity (192, 208, 209).

## 7 Conclusion and perspectives

### 7.1 Major research advances

Single-cell, spatial and immunogenomic studies have improved resolution of NEPC-associated ecosystems, but longitudinal human sampling remains insufficient to distinguish cause, adaptation and correlation. A temporal model is emerging: androgen-receptor pathway pressure permits epithelial state transition; tumor and stromal cytokine programs, macrophage/CAF remodeling and hypoxia may then stabilize selected states; immune dysfunction and treatment tolerance can follow. Each step is supported at a different evidence level and should not be assumed to occur in every patient.

Recent atlases and lineage studies provide testable markers for this sequence (19, 29, 36, 43–45). Paired pre-treatment, transition-state and post-NEPC biopsies are needed to establish directionality and to identify windows for intervention.

Human data support heterogeneous immune depletion, FAP-associated stromal features and DLL3 expression in subsets, while many CAF, ECM, vascular and metabolic mechanisms remain model-based or extrapolated (18–20, 31, 33). These distinctions are summarized in **Tables 1**–**3**.

**Table 3:** Evidence levels, representative references and biomarker roles for NEPC-TME hallmarks and therapeutic opportunities.

Component/strategy	Evidence level and representative references	Evidence basis	Clinical interpretation/intended biomarker use
DLL3-directed therapy	Level A/B (31, 34, 183, 184)	Human expression data; phase 1b tarlatamab; preclinical radiopharmaceutical models.	Early clinical/preclinical. DLL3 is a patient-selection marker; expression is not a universal NEPC feature.
CAF/FAP/stromal remodeling	Level A/C/D (19, 33, 49, 167, 168)	FAP expression and human stromal associations; much therapeutic rationale remains extrapolated.	FAP is a candidate prognostic, imaging, and trial-selection marker. Expression alone does not predict benefit from FAP-directed therapy.
Myeloid, Treg, and MDSC programs	Level A/C/D (19, 20, 36, 69, 70, 128, 129)	Human immunogenomic/scRNA observations plus prostate-cancer and pan-cancer mechanisms.	Supports immune-contexture stratification and pharmacodynamic studies; NEPC-specific combination trials are lacking.
ECM stiffness/fibrosis	Level B/C/D (39, 41, 109, 113–115, 117–119)	NEPC-relevant models, prostate-cancer mechanobiology, and general collagen-barrier literature.	Candidate mechanism only. Direct quantitative collagen, pressure, and drug-distribution data in human NEPC are needed.
Vascular remodeling/hypoxia	Level B/C/D (10, 89, 94, 148–150)	NEPC-relevant angiogenic models plus broader vascular-normalization literature.	Potential pharmacodynamic imaging domain; NEPC permeability, perfusion, and drug-distribution pharmacology remain unvalidated.
Metabolic/epigenetic lineage plasticity	Level B, with Level C stromal lactate evidence (42–48)	Tumor-intrinsic NEPC models; separate prostate-cancer CAF-lactate studies.	Promising preclinical vulnerabilities, not TME-only strategies. Biomarkers remain exploratory and mechanism-specific.

Levels map directly to **Table 1**. Representative references are included for transparency; the ranking is an evidence synthesis, not a clinical guideline recommendation.

The TME may contribute to resistance to immunotherapy, chemotherapy and targeted agents through immune contexture, survival signals, tissue mechanics and metabolic constraints. Direct demonstration of necessity, sufficiency or therapeutic reversal in NEPC is uncommon, so these pathways are best viewed as candidate mechanisms and biomarker hypotheses.

### 7.2 Future research directions

Future research should link longitudinal spatial multi-omics, faithful humanized and organoid-immune-stroma models, perturbation-based validation, and biomarker-integrated trials.

Longitudinal spatial multi-omics should connect epithelial state changes with macrophage, CAF, endothelial and lymphoid remodeling in the same patients (19, 37, 210). Sampling before lineage transition, during mixed states and after histologic NEPC would clarify sequence and reversibility.

Humanized mice, patient-derived organoids and immune-stroma co-cultures can test causality, but model fidelity must be documented for lineage state, immune composition and spatial architecture (202–205).

Systems approaches may integrate these measurements and nominate interactions for perturbation, but computational association should be followed by source-resolved and intervention-based validation (182, 211, 212).

Biomarker-integrated trials should prespecify intended use: DLL3 or other targets for patient selection; baseline FAP or immune contexture as candidate prognostic factors; treatment-by-marker interactions for predictive claims; and serial circulating, imaging or spatial measures for pharmacodynamic monitoring. Empiric combinations without this distinction are unlikely to resolve which TME mechanism, if any, is clinically actionable.

Additional studies support this research agenda. Metabolic adaptation, tumor–stroma lactate and glycolytic interactions, metabolic immunosuppression, and candidate lactate-targeting approaches have been reported in prostate cancer and related tumor models (213–219). STAT3-associated signaling and androgen receptor-active to NEPC reprogramming provide additional mechanistic context for lineage plasticity (220, 221). Vascular response monitoring and engineered vascular leakiness are relevant to future pharmacodynamic and drug-delivery studies (222, 223).
